# Transient cell assembly networks encode stable spatial memories

**DOI:** 10.1038/s41598-017-03423-3

**Published:** 2017-06-21

**Authors:** Andrey Babichev, Yuri Dabaghian

**Affiliations:** Department of Computational and Applied Mathematics, Rice University, Houston, TX 77005 USA

## Abstract

One of the mysteries of memory is that it can last despite changes in the underlying synaptic architecture. How can we, for example, maintain an internal spatial map of an environment over months or years when the underlying network is full of transient connections? In the following, we propose a computational model for describing the emergence of the hippocampal cognitive map in a network of transient place cell assemblies and demonstrate, using methods of algebraic topology, how such a network can maintain spatial memory over time.

## Introduction

The mammalian hippocampus plays a major role in spatial cognition by producing an internalized representation of space, or a cognitive map of the environment^1–4^. Several key observations shed light on the neuronal computations responsible for implementing such a map. The first observation is that the spiking activity of the principal cells in the hippocampus is spatially tuned. In rats, these neurons, called place cells, fire only in certain locations within the environment—their respective place fields^5^. As demonstrated in many studies, this simple principle allows us to map the animal’s ongoing trajectory^6, 7^, its past navigational experience^8^, and even its future planned routes^9–11^ from the place cell’s spiking activity.

The second observation is that the spatial layout of the place fields—the place field map—is flexible: as the environment is deformed, the place fields shift and change their shapes, while preserving their mutual overlaps, adjacency and containment relationships^12–15^. Thus, the sequential order of place cells’ (co)activity induced by the animal’s moves through a morphing environment remains invariant within a certain range of geometric transformations^16–20^. This implies that the place cells’ spiking encodes a coarse framework of qualitative spatiotemporal relationships, such that the hippocampal map provides a ready topological framework which can be filled in with more detailed metrical data input by other brain regions.

The third observation concerns the synaptic architecture of the (para)hippocampal network: it is believed that groups of place cells that demonstrate repeated coactivity form functionally interconnected cell assemblies, which together drive their respective “reader-classifier” or “readout” neurons in the downstream networks^21, 22^. The activity of a readout neuron actualizes the qualitative relationships between the regions encoded by the individual place cells, thus defining the type of spatial connectivity information encoded in the hippocampal map^23^.

A given cell assembly network architecture appears as a result of spatial learning, i.e., it emerges from place cell coactivities produced during an animal’s navigation through a particular place field map, via a “fire-together-wire-together” plasticity mechanism^24, 25^. A salient property of the cell assemblies is that they may disband as a result of a depression of synapses caused by reduction or cessation of spiking activity over a sufficiently long timespan^26^. Some of the disbanded cell assemblies may later reappear during a subsequent period of coactivity, then disappear again, and so forth. Electrophysiological studies suggest that the lifetime of the cell assemblies ranges between minutes^27, 28^ and hundreds of milliseconds^29–33^. In contrast, spatial memories in rats can last much longer^34–36^, raising the question: how can a large-scale spatial representation of the environment be stable if the neuronal substrate changes on a much shorter timescale?

The hypothesis that the hippocampus encodes a topological map of the environment allows us to address this question computationally, using methods derived from the field of algebraic topology. Below, we propose a phenomenological model of a transient hippocampal network and use persistent homology theory^37–39^ to demonstrate that a large-scale topological representation of the environment encoded by this network can remain stable despite the transience of neuronal connections.

## The Model

We use a computational model to integrate the information provided by individual place cells into a large-scale topological representation of the environment; we have described this model in detail elsewhere^40–44^ but briefly outline it here. Alexandrov^45^ and Čech^46^ noted that if one covers a space *X* with a sufficient number of regions *U*_1_, *U*_2_, …, *U*_*n*_, then it is possible to reconstruct the topology of *X* from the pattern of overlaps between these regions. To do that, one builds what is known as a “nerve simplicial complex” or simply “nerve of the cover” ${\mathscr{N}}$: each element *U*_*i*_ defines a vertex of ${\mathscr{N}}$, each pair of overlapping elements, *U*_*i*_ and *U*_*j*_, defines a 1*D* simplex (a bond), *σ*_*ij*_ and so on. The Alexandrov-Čech theorem states that if every such overlap is contractible in *X*, then the nerve of the cover, ${\mathscr{N}}$, is topologically equivalent to the covered space *X* ^47^. If *X* is viewed as the environment and *U*_*i*_’s as the place fields, then this construction suggests that the place field map encodes the topological information of the space navigated by the rat.

One can visualize the process of building the nerve simplicial complex (i.e., learning a space) as follows: as soon as an animal enters a place field *U*_*i*_, the simplicial complex ${\mathscr{N}}$ acquires a vertex *v*_*i*_; as soon as the intersection of two place fields is visited, a link, *σ*_*ij*_, appears between the vertices *v*_*i*_ and *v*_*j*_. Visiting a location where three place fields overlap contributes a triangle, *σ*_*ijk*_, between vertices *v*_*i*_, *v*_*j*_ and *v*_*k*_, and so on (see Suppl. Fig. 1). As the animal explores the environment, new overlaps are detected and the nerve simplicial complex grows over time, ${\mathscr{N}}={\mathscr{N}}(t)$. If the environment is densely covered with place fields, then there will be a moment, *T*_*min*_, when the animal will have sampled a critical mass of the intersections between the place fields, at which point the simplicial complex ${\mathscr{N}}({T}_{min})$ will be sufficiently dense to capture the topological structure of the underlying environment.

One might be tempted to view this argument as a mathematical proof that the information encoded in place cell activity is sufficient to encode the topological features of the environment, but the reality is more complex: we must remember that place fields are artificial constructs used by experimentalists to visualize the spike data. The hippocampus and the downstream brain regions do not have access to the elements of the geometric construction described above—the shape of the place fields or their locations. For the brain, the information is represented only by place cell spiking activity: if the animal enters a location where several place fields overlap, this fact will be detected by the downstream brain areas only by sensing the co-firing of the respective place cells. In other words, physical overlap of place fields necessitates temporal overlap of the firing of the respective place cells.

**Figure 1:**
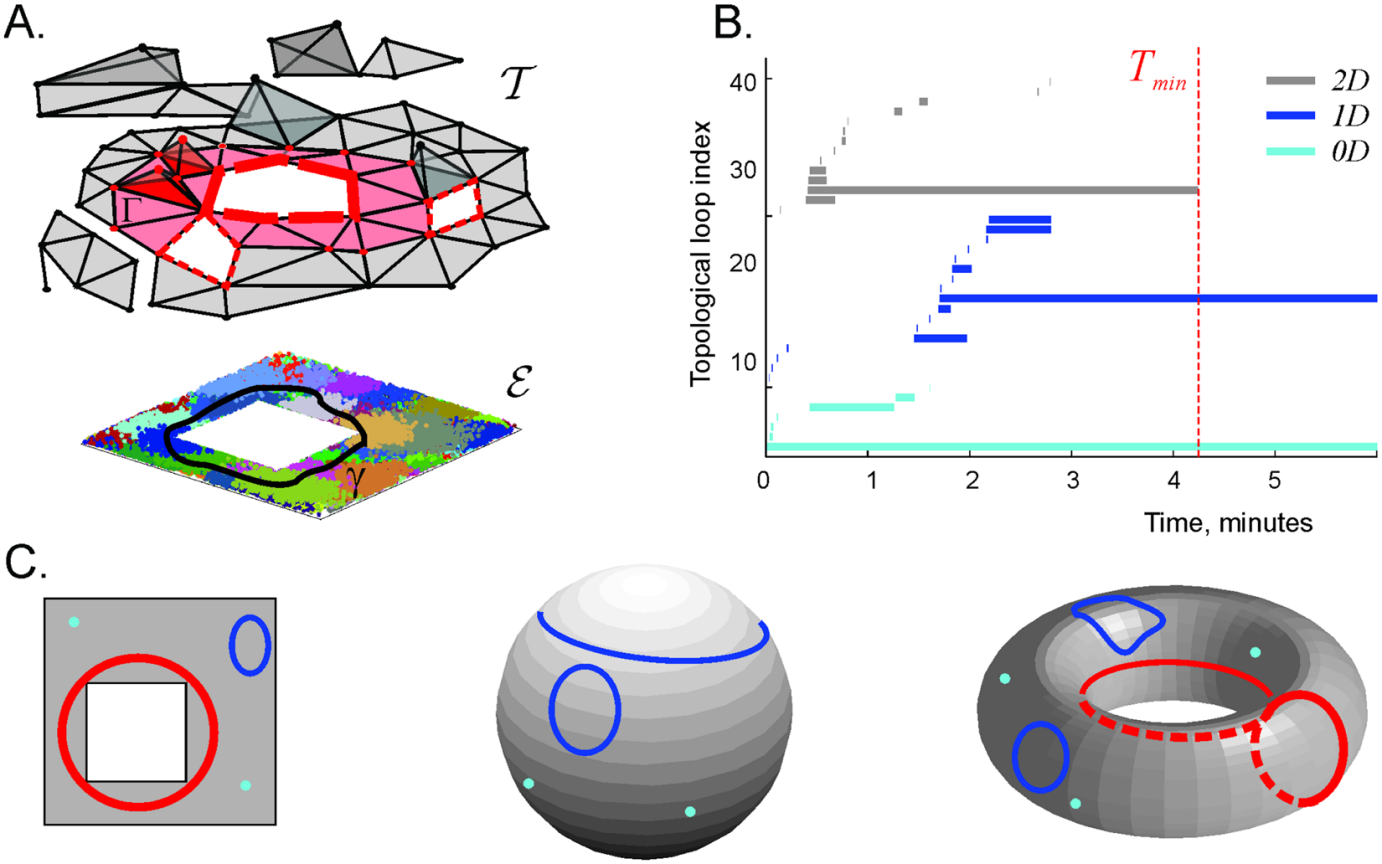
The place cell coactivity complex. (**A**) A simulated rat traverses an environment ${\mathcal E}$ with a hole in the middle, covered by place fields (colored regions). Areas where place fields overlap imply place cell co-firing; this is represented by the coactivity complex ${\mathscr{J}}$. Vertices of ${\mathscr{J}}$ correspond to active neurons, links to pairs of coactive neurons, etc. Early in exploring the space, the complex contains gaps, or transient topological noise, that are filled in with further exploration over time. The hole in the middle of ${\mathscr{J}}$ corresponds to the central hole in ${\mathcal E}$. The non-contractible closed chain of pink simplexes represents the cell assemblies ignited along one path the animal traverses (loop in ${\mathcal E}$). (**B**) Timelines of zero-dimensional (0*D*, cyan), one-dimensional (1*D*, blue) and two-dimensional (2*D*, gray) topological loops in ${\mathscr{J}}$. The number of 0*D*, 1*D* and 2*D* lines at every moment defines the Betti values, *b*_0_, *b*_1_ and *b*_2_. As long as a given 0*D* loop persists, ${\mathscr{J}}$ contains the corresponding connected space (a gray triangle in panel A), a 1*D* loop represents a noncontractible hole and a 2*D* loop represents a bubble in ${\mathscr{J}}$. In the illustrated case, the 0*D* spurious loops disappear in 1.5 mins, when ${\mathscr{J}}$ fuses into one piece. The spurious 1*D* loops disappear in about 2.8 min, when all the spurious holes in ${\mathscr{J}}$ close up, and the 2*D* loops disappear by *T*_*min*_ = 4.2 min, at which point ${\mathscr{J}}$ becomes topologically equivalent to ${\mathcal E}$. (**C**) The barcode: topological loops in the environment ${\mathcal E}$, sphere *S* and torus *T*. In all three cases, any two 0*D* loops (i.e., points) can be matched with one another via continuous displacements. Thus, all 0*D* loops are topologically equivalent to a single representative 0*D* loop, i.e., ${b}_{0}( {\mathcal E} )={b}_{0}(S)={b}_{0}(T)=1$. The 1*D* loops are of two types: some contract to a point (blue loops), others are non-contractible due to topological obstructions, e.g., the central hole in ${\mathcal E}$. Thus, ${b}_{1}( {\mathcal E} )=1$, *b*_1_(*S*) = 0 and *b*_1_(*T*) = 2. Since the sphere and the torus are 2*D* surfaces that loop onto themselves, their second Betti numbers are *b*_2_(*S*) = *b*_2_(*T*) = 1. ${\mathcal E}$ is also 2*D* but it can be contracted to the 1*D* rim of the central hole, hence ${b}_{2}( {\mathcal E} )=0$. Lastly, none of the three shapes extend beyond 2*D*, so there are no higher-order Betti numbers. The barcodes are ${\mathfrak{b}}( {\mathcal E} )=(1,1,0,0,\ldots )$, ${\mathfrak{b}}(S)=\mathrm{(1},0,1,0,0,\ldots )$, and ${\mathfrak{b}}(T)=\mathrm{(1},2,1,0,0,\ldots )$.

The rest of the construction is similar: the activity of a place cell, *c*_*i*_, is represented by a vertex *v*_*i*_; the detected coactivity of two place cells, *c*_*i*_ and *c*_*j*_—by a bond, *σ*_*ij*_, between vertices *v*_*i*_ and *v*_*j*_; the detected coactivity of three place cells, *c*_*i*_, *c*_*j*_ and *c*_*k*_—by a 2*D* simplex *σ*_*ijk*_ and so on. This procedure will produce a temporal “coactivity complex” ${\mathscr{J}}(t)$ that is analogous to the ${\mathscr{N}}(t)$ constructed above: at every moment of time *t*, the coactivity complex ${\mathscr{J}}(t)$ represents only those place cell combinations that have exhibited (co)activity. As the animal begins to explore the environment, the newly emerging coactivity complex is small, fragmented and contains many holes, most of which do not correspond to physical obstacles or to the regions that have not yet been visited by the animal (Fig. 1A). These gaps tend to disappear as the pool of place cell coactivities accumulates. Numerical simulations show that, if place cells operate within biological parameters^40^, the topological structure of ${\mathscr{J}}$ becomes equivalent to the topological structure of the environment within minutes. The minimal time *T*_*min*_ required to produce a correct topological representation of the environment can then be used as an estimate for the time required by a given place cell ensemble to learn the topological structure or spatial connectivity of the experimental environment (Fig. 1B, refs 40–44), and the coactivity complex ${\mathscr{J}}(t)$ itself may serve as a schematic model of the hippocampal map^23^. The simplexes of ${\mathscr{J}}(t)$, just like the individual cell groups, provide local information about the environment, but together, as a “coactivity” simplicial complex, they represent space as a whole, providing a link between the cellular and the net systemic level of the information processing.

### The large-scale topology

The topological structure of a space *X* can be described in terms of the topological loops that it contains, i.e., in terms of its non-contractible surfaces counted up to topological equivalence. A more basic topological description of *X* is provided by simply counting the topological loops in different dimensions, i.e., by specifying its Betti numbers *b*_*n*_(*X*)^47^. The list of the Betti numbers of a space *X* is known as its topological barcode, ${\mathfrak{b}}(X)=({b}_{0}(X),{b}_{1}(X),{b}_{2}(X),\ldots )$, which in many cases captures the topological identity of topological spaces^37^. For example, the environment ${\mathcal E}$ shown at the bottom of Fig. 1A has the topological barcode ${\mathfrak{b}}( {\mathcal E} )=(1,1,0,\ldots )$, which implies that ${\mathcal E}$ is topologically equivalent to an annulus (Fig. 1C). Other familiar examples of topological shapes identifiable via their topological barcodes are a two-dimensional sphere *S* and a torus *T* with the barcodes ${\mathfrak{b}}(S)=(1,0,1,0,\ldots )$ and ${\mathfrak{b}}(T)=\mathrm{(1},2,1,0,\ldots )$ respectively (Fig. 1C). For the mathematically oriented reader, we note that the matching of topological barcodes does not always imply topological equivalence between topological spaces but, in the context of this study, we disregard effects related to torsion and other topological subtleties.

The analyses of the coactivity complex is based on comparing its topological barcode ${\mathfrak{b}}({\mathscr{J}})$ to the topological barcode of the environment, ${\mathfrak{b}}( {\mathcal E} )$. If these barcodes do not match, then ${\mathscr{J}}$ and ${\mathcal E}$ are topologically distinct. In contrast, if the barcode of ${\mathscr{J}}$ is “physical,” i.e., coincides with ${\mathfrak{b}}( {\mathcal E} )$, then the coactivity complex provides a faithful representation of the environment. More conservatively, one may compare only the physical dimensions in the barcodes ${\mathfrak{b}}({\mathscr{J}})$ and ${\mathfrak{b}}( {\mathcal E} )$, i.e., 0*D*, 1*D*, 2*D* loops, or the dimensions containing the nontrivial 0*D* and 1*D* loops for the environment, as shown on Fig. 1B.

### Simplicial model of the hippocampal network

**Figure 2:**
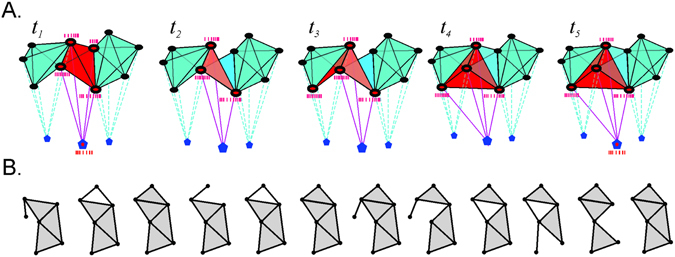
Place cell assemblies and flickering coactivity complexes. (**A**) Functionally interconnected groups of place cells (place cell assemblies) are schematically represented by fully interconnected cliques. The place cells (small disks) in a given assembly *ς* are synaptically connected to the corresponding readout neuron *n*_*ς*_ (pentagons below). An assembly *ς* ignites (red clique/tetrahedron in the middle) when its place cells elicit jointly a spiking response from the readout neuron *n*_*ς*_ (active cells have red centers). A cell assembly may be active at a certain moment of time, then deactivate, then become active again, and so forth. If a certain cell assembly ceases to ignite and another combination of place cells begins to exhibit frequent coactivity, the old cell assembly is replaced by new one. (**B**) Formation and disbanding of the cell assemblies in the hippocampal network is schematically represented by appearance and disappearance of the maximal simplexes of the “flickering” coactivity complex. The sequence of figures illustrates fluctuations in a small part of the coactivity complex shown on Fig. 1A. Since the large-scale topological information encoded by the hippocampal network should persist, one would expect that the flickering complex should retain a certain skeletal structure, capable of representing such information.

If every observed group of coactive place cells contributes a simplex, the resulting coactivity complex ${\mathscr{J}}$ makes no reference to the structure of the hippocampal network, and gives a purely phenomenological description of the information contained in the place cell coactivity. In a more detailed approach, the coactivity complex may be constructed so that its maximal simplexes (i.e., the simplexes that are not subsimplexes of any larger simplex) represent ignitions of the place cell assemblies, rather than arbitrary place cell combinations. The combinatorial arrangement of the maximal simplexes in the resulting “cell assembly coactivity complex,” denoted ${{\mathscr{J}}}_{CA}$, schematically represents the network of interconnected cell assemblies^23, 42^ (Fig. 2A).

The specific algorithm for constructing the complex ${{\mathscr{J}}}_{CA}$ may also reflect how neuronal coactivity is processed by the readout neurons. If these neurons function as “coincidence detectors,” i.e., if they react to the spikes received within a short coactivity *detection* period *w*_*σ*_ (typically, *w*_*σ*_ ≈ 200–250 milliseconds^41, 48^), then the maximal simplexes *σ* in the corresponding coincidence detection coactivity complex (denoted ${{\mathscr{J}}}_{\sigma }$) will appear instantaneously at the moments of the cell assemblies’ ignitions^42, 43^. Alternatively, if the readout neurons integrate the coactivity inputs from smaller parts of their respective assemblies over an extended coactivity *integration* period *ϖ*_*ς*_^49, 50^, then the appearance of a maximal simplex *ς* in the corresponding input integration coactivity complex (denoted as ${{\mathscr{J}}}_{\varsigma }$) will extend over time, reflecting the dynamics of synaptic integration. Physiologically, *ϖ*_*ς*_ can be viewed as the period during which the readout neuron *n*_*ς*_ connects synaptically to a particular combination of coactive place cells, forming a cell assembly *ς*, retaining these connections, responding to subsequent ignitions of *ς* and maintaining information of which cells spiked together—this is the readout neuron’s “finite memory span.” Clearly, the distribution of the parameters *ϖ*_*ς*_ in a given cell assembly network affects the rate at which large-scale topological information is accumulated and thus controls the model’s description of spatial learning.

To model an input integrator coactivity complex ${{\mathscr{J}}}_{\varsigma }$, we first built a relational graph *G* that represents the pool of the most coactive pairs of place cells. Specifically, for every cell *c*_*i*_, we select *n*_0_ cells *c*_*j*_, *j* = 1, …, *n*_0_, that exhibit the highest rates ${f}_{{c}_{i},{c}_{j}}$ of coactivity with *c*_*i*_. The maximal, fully interconnected subgraphs of the graph *G*—its cliques—can then be then be identified with the cell assemblies and viewed as simplexes *ς* of the clique complex ${{\mathscr{J}}}_{\varsigma }(G)$ ^51^. The process of assembling the cliques from pairwise connections can be used to model the process of integrating spiking inputs in the cell assemblies, so that the resulting clique coactivity complex ${{\mathscr{J}}}_{\varsigma }$ serves as a model of the input integration cell assembly network. Numerical simulations show that the input integration clique complexes reproduce the topological structure of the environment faster and more reliably than coincidence detection coactivity complexes ${{\mathscr{J}}}_{\ast }={{\mathscr{J}}}_{\sigma }$ ^23, 42, 43^. In fact, the coincidence detection coactivity complexes can be viewed as a specific case of the input integration coactivity complexes: as the integration period shrinks and approaches the coactivity period *ϖ*_*ς*_ → *w*_*σ*_, the input integration coactivity complex ${{\mathscr{J}}}_{\varsigma }$ reduces to the coincidence complex ${{\mathscr{J}}}_{\sigma }$. For all these reasons we will model only the input integration, i.e., clique coactivity complexes.

### Instability of the cell assemblies

In the foregoing construction of the coactivity complexes ${{\mathscr{J}}}_{\varsigma }$, a given cell assembly could take the entire navigation period to form *ϖ*_*ς*_ = *T*_*tot*_. It is then presumed to exist from the moment of its first appearance for as long as the navigation continues^42^. A natural approach to modeling cell assemblies with finite lifetimes is to restrict the period during which they can form, so that *ϖ*_*ς*_ < *T*_*tot*_. In a population of cell assemblies, the integration periods can be distributed with a certain mode *ϖ* and a variance Δ_*ϖ*_. In order to simplify the approach, we will make two assumptions. First, we will describe the entire population of the readout neurons in terms of the integration period of a typical readout neuron, describing the ensemble of readout neurons with a single parameter, *ϖ*. Second, we will assume that the integration periods of all neurons are synchronized, i.e., that there exists a globally defined coactivity integration window of width *ϖ* during which the entire population of the readout neurons synchronously processes coactivity inputs from their respective place cell assemblies. In such case, *ϖ* can be viewed as a period during which the cell assembly network processes the ongoing place cell spiking activity. Below we demonstrate that these restrictions result in a simple model that allows us to describe a population of finite lifetime cell assemblies and show that the resulting cell assembly network, for a sufficiently large *ϖ*, reliably encodes the topological connectivity of the environment.

### Computational model of the transient cell assembly network

To build a coactivity complex ${\mathcal F}$ with fluctuating or “flickering” maximal simplexes that represents a network with rewiring cell assemblies, we implement a “sliding coactivity integration window” approach (see Suppl. Movie 1). First, we identify the maximal simplexes that emerge within the first *ϖ*-period after the onset of the navigation, *ϖ*_1_, based on the place cell activity rates evaluated within that window, *f*_*ς*_(*ϖ*_1_), and construct the corresponding input integration coactivity complex ${\mathcal F} ({\varpi }_{1})$. Then the algorithm is repeated for the subsequent windows *ϖ*_2_, *ϖ*_3_, … which are obtained by shifting the starting window *ϖ*_1_ over small time steps Δ*t*. Since consecutive windows overlap, the corresponding coactivity complexes ${\mathcal F} ({\varpi }_{1})$, ${\mathcal F} ({\varpi }_{2})$, … consist of overlapping sets of maximal simplexes. A given maximal simplex *ς* (uniquely defined by the set of its vertexes in any window) may appear in a chain of consecutive windows *ϖ*_1_, *ϖ*_2_, …, *ϖ*_*k* − 1_ then disappear at a step *ϖ*_*k*_ (i.e., $\varsigma \in  {\mathcal F} ({\varpi }_{k-1})$, but $\varsigma \notin  {\mathcal F} ({\varpi }_{k})$), then reappear in a later window *ϖ*_*l*_, then disappear again, and so forth (Fig. 2B). The midpoint *t*_*k*_ of the window in which the maximal simplex *ς* has (re)appeared defines the moment of *ς*’s (re)birth, and the midpoints of the windows where it disappears, are viewed as the times of its deaths. Indeed, one may use the left or the right end of the shifting integration window, which would affect the endpoints of the navigation, but not the net results discussed below. As a result, the lifetime *δt*_*ς*,,*k*_ of a cell assembly *ς* between its *k*-th consecutive appearance and disappearance can be as short as one discrete time step Δ*t* (if *ς* appears within a window *ϖ*_*k*_ and disappears at the next step, within *ϖ*_*k* + 1_), or as long as *T*_*tot*_ - *ϖ* in the case if *ς* appears at the first step and never disappears. However, a typical maximal simplex exhibits a spread of lifetimes that can be characterized by a half-life, as we will discuss below.

It is natural to view the coactivity complexes ${\mathcal F} ({\varpi }_{i})$ as instances of a single *flickering coactivity complex* ${ {\mathcal F} }_{\varpi }$, ${\mathcal F} ({\varpi }_{i})={ {\mathcal F} }_{\varpi }({t}_{i})$, with appearing and disappearing maximal simplexes (Fig. 2B). In the following, we will use ${ {\mathcal F} }_{\varpi }$ as a model of the transient cell assembly network and study whether such a network encodes a correct and stable topological map of the environment on a moment-by-moment basis.

## Results

### Flickering cell assemblies

**Figure 3:**
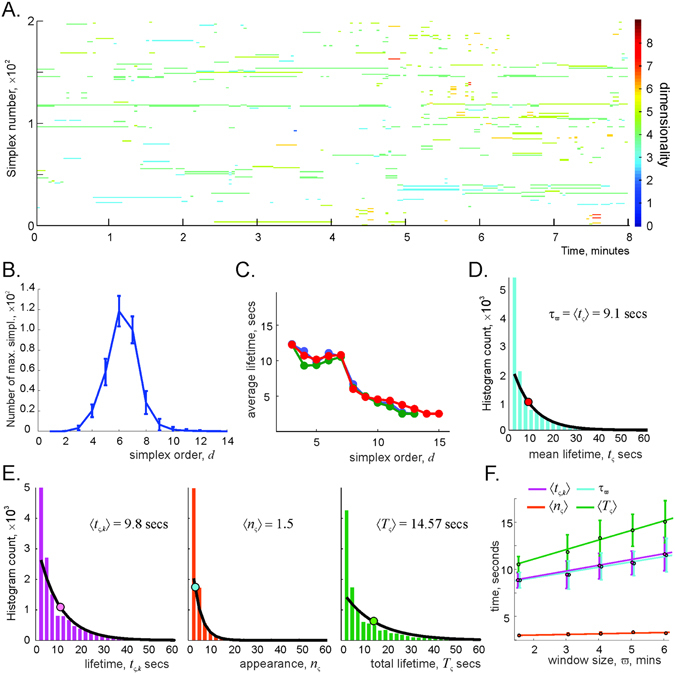
Fluctuating simplexes. (**A**) Each strike represents a timeline of a particular maximal simplex *ς*, computed for the coactivity window *ϖ* = 4 min. There are about *N*_*ς*_ = 320 maximal simplexes at every given timestep (first 200 are enumerated along the *y*-axis), whereas the total number of maximal simplexes observed during the entire navigation period is about 11,000. The color of the timelines marks the order of *ς* (colorbar on the right). Notice that the simplexes of lower orders generally persist over longer intervals. (**B**) Number of maximal simplexes as a function of their order has a Gaussian shape with the mean *d* = 7 and standard deviation Δ_*d*_ ≈ ±2, suggesting that a typical cell assembly contains about seven neurons and about two neurons may appear or disappear from it at a given moment. (**C**) Average existence time of the maximal simplexes tends to decay with increasing order. (**D**) Histogram of the maximal simplexes’ individual average lifetimes *t*_*ς*_ fit with the exponential distribution with mean *τ*_*ϖ*_ = 9 s, defining the half-life of the simulated cell assemblies for this *ϖ*. (**E**) Histogram of the maximal simplexes’ lifetimes *t*_*ς*,,*k*_, i.e., of the lengths of all intervals between consecutive appearance and disappearance of the maximal simplexes, the histogram of the number of simplex-births *n*_*ς*_ and the histogram of the total existence periods *T*_*ς*_ fit with their respective exponential distributions. The mean number of simplex’ appearances 〈*n*_*ς*_〉 ≈ 1.5 shows that most maximal simplexes appear only once or twice, though some maximal simplexes may appear 20 times or more. Notice that the mean net existence period 〈*T*_*ς*_〉 ≈ 14.57 s is approximately equal to the product of the mean lifetime and the mean number of appearances 〈*T*_*ς*_〉 ≈ 〈*n*_*ς*_〉 〈*t*_*ς*,,*k*_〉. (**F**) As the size of the memory window *ϖ* increases, the lifetimes, half-lives, and net existence periods of the maximal simplexes grow linearly with *ϖ*.

We studied the dynamics of flickering cell assemblies produced by a neuronal ensemble containing *N*_*c*_ = 300 simulated place cells. First, we built a simulated cell assembly network as described above that contains, on average, about *N*_*ς*_ ≈ 320 finite lifetime, or transient, cell assemblies (Fig. 3A). As shown in Fig. 3B, the order of the maximal simplexes that represent these assemblies, ranges between |*ς*| = 2 and |*ς*| = 14, with the mean of about |*ς*| = 7, implying that a typical simulated cell assembly includes |*ς*| = 7 ± 2 cells.

The distribution of the maximal simplexes’ lifetimes *δt*_*ς*,,*k*_ as a function of their dimensionality shows that higher-dimensional simplexes (and thus the higher-order cell assemblies) are shorter-lived than the low-order cell assemblies (Fig. 3C). The histogram of the mean lifetimes *t*_*ς*_ = 〈*δt*_*ς*,,*k*_〉_*k*_ is closely approximated by the exponential distribution (Fig. 3D), which suggests that the duration of the cell assemblies’ existence can be characterized by a half-life *τ*_*ϖ*_. The individual lifetimes *δt*_*ς*,*k*_, the number of appearances *n*_*ς*_, and net existence time Δ*T*_*ς*_ = ∑_*k*_ *δt*_*ς*,,*k*_ of the maximal simplexes are also exponentially distributed (see Fig. 3E and Suppl. Fig. 2). As expected, the mean net existence time approximately equals the product of the mean lifetime and the mean number of the cell assembly’s appearances 〈Δ*T*_*ς*_〉 ≈ 〈*n*_*ς*_〉 〈*δt*_*ς*,,*k*_〉.

Figure 3F shows how these parameters depend on the width of the integration window. As *ϖ* widens, the mean lifetime *t*_*ς*_ of maximal simplexes (and thus its half-life and the net lifetime) grows linearly, whereas the mean number of simplexes’ appearances 〈*n*_*ς*_〉 remains nearly unchanged. The latter result is natural since the frequency with which the cell assemblies ignite is defined by how frequently the animal visits the respective cell assembly fields (the domains where the corresponding sets of place fields overlap^42^). This frequency does not change significantly if the changes in *ϖ* do not exceed the characteristic time required to turn around the maze and revisit cell assembly fields, in this case ca. 1–2 min. Thus, the model produces a population of rapidly changing cell assemblies; in the simulated case *τ*_*ϖ*_ ≈ 9 seconds, which is close to the experimental range of values^22^. These results allow us to address our main question: can a network of transient cell assemblies encode the topology of the environment?

### Flickering coactivity complex

**Figure 4:**
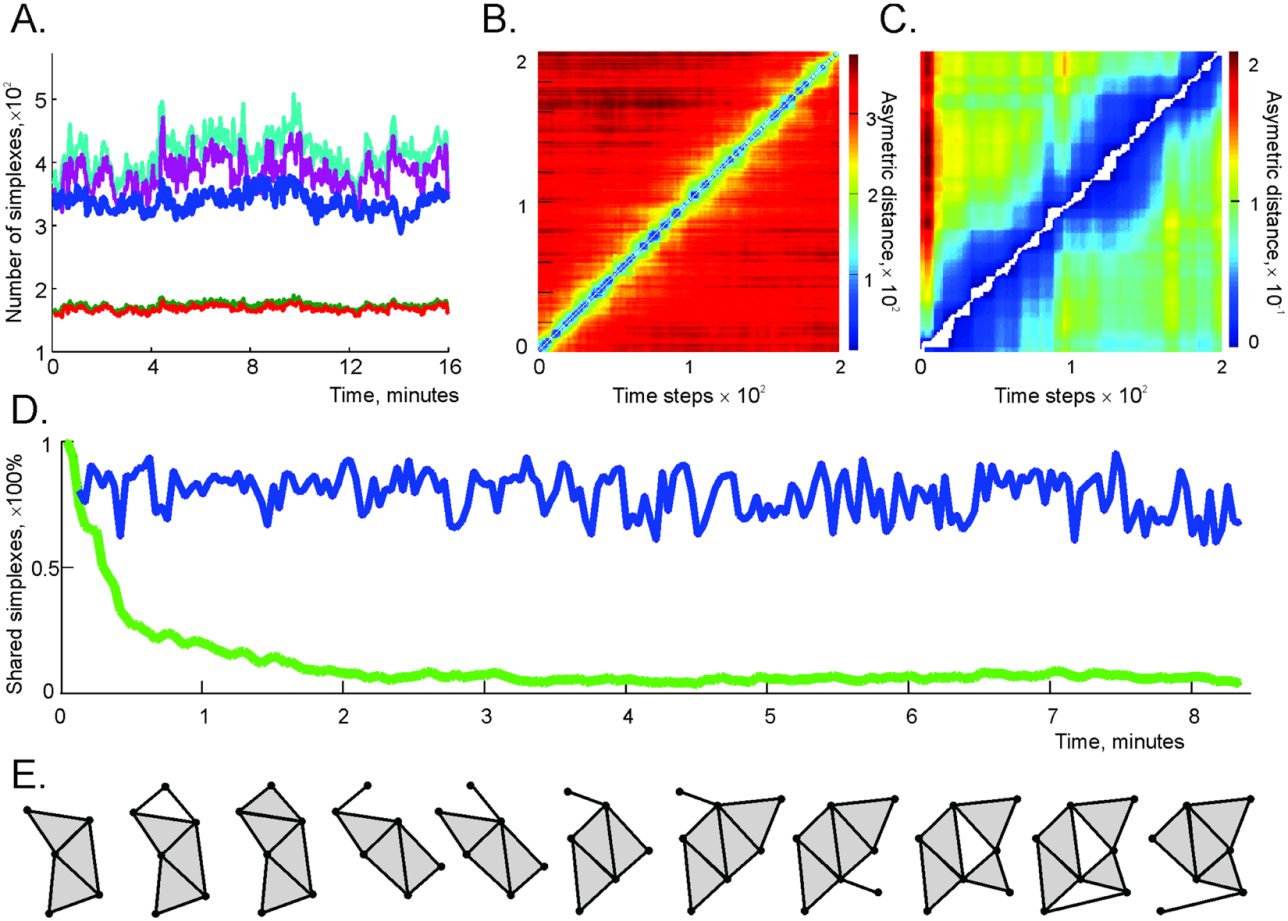
Behavior of the flickering coactivity complex computed for the memory window width *ϖ* = 4 min, shifted over Δ*t* = 2.5 secs steps, i.e., at the discrete times *t*_*k*_ = *k*Δ*t*. (**A**) The number of maximal simplexes in ${ {\mathcal F} }_{\varpi }$ (blue trace) fluctuates within 4% of the mean value of *N*_*ς*_ = 320. The number of the 1*D* simplexes ${N}_{1}({ {\mathcal F} }_{\varpi })$ (red trace) and the number of the 1*D* simplexes appearing in consecutive windows (i.e., links shared by ${ {\mathcal F} }_{\varpi }({t}_{i})$ and ${ {\mathcal F} }_{\varpi }({t}_{i-1})$, green trace) fluctuate within a 3% bound. The fluctuations in the number of 2*D* subsimplexes (${N}_{2}({ {\mathcal F} }_{\varpi })$, light blue trace) and the number of 2*D* simplexes shared by two consecutive windows (purple trace) do not exceed 7% of the mean. ${N}_{1}({ {\mathcal F} }_{\varpi })$ and ${N}_{2}({ {\mathcal F} }_{\varpi })$ are scaled down by a factor of 10 to fit the scale of the figure. (**B**) The asymmetric distance *d*_*ij*_ between ${ {\mathcal F} }_{\varpi }({t}_{i})$ and ${ {\mathcal F} }_{\varpi }({t}_{j})$ is defined as the number of the maximal simplexes at moment *t*_*i*_ that are missing at the moment *t*_*j*_. As the timestep separation |*i* − *j*| grows, *d*_*ij*_ rapidly increases. (**C**) The matrix of similarity coefficients *r*_*ij*_ between the weighted coactivity graphs at different timesteps. For close timesteps *i* and *j*, the differences between the corresponding coactivity graphs *G*(*t*_*i*_) and *G*(*t*_*j*_) are small, but as time separation grows, the differences accumulate, though not as rapidly as with the coactivity complexes. (**D**) At each moment, *t*_*i*_, the blue line shows the proportion of maximal simplexes present at the previous time, *t*_*i* − 1_. The green line shows the proportion of maximal simplexes contained at the start (in ${ {\mathcal F} }_{\varpi }({t}_{1})$) that remain in the coactivity complex at the later time ${ {\mathcal F} }_{\varpi }({t}_{i})$. The population of simplexes changes by about 95% in about 2 min. (**E**) A schematic illustration of the changes of the coactivity complex’s shape: rather than exhibiting fluctuations around a certain “mean” shape (Fig. 2B), the coactivity complex continuously restructures.

We next studied the properties of the flickering coactivity complex ${ {\mathcal F} }_{\varpi }$ formed by the pool of fluctuating maximal simplexes. First, we observed that the size of ${ {\mathcal F} }_{\varpi }$ does not fluctuate significantly across the rats’ navigation time. As shown in Fig. 4A, the number of maximal simplexes ${N}_{\varsigma }({ {\mathcal F} }_{\varpi }(t))$ fluctuates within about 4% of its mean value. The fluctuations in the number of coactive pairs ${N}_{2}({ {\mathcal F} }_{\varpi }(t))$ is even smaller: 3% of the mean, and the variations in number of the third order simplexes ${N}_{3}({ {\mathcal F} }_{\varpi }(t))$ are about 7% of the mean. To quantify the structural changes in ${ {\mathcal F} }_{\varpi }$, we computed the number of maximal simplexes that are present at time *t*_*i*_ and missing at time *t*_*j*_, yielding the matrix of asymmetric distances, ${d}_{ij}={N}_{\varsigma }({ {\mathcal F} }_{\varpi }({t}_{i})\backslash { {\mathcal F} }_{\varpi }({t}_{j}))$ for all pairs *t*_*i*_ and *t*_*j*_ (see Methods and Fig. 4B). The result suggests that as temporal separation |*t*_*i*_ − *t*_*j*_| increases, the differences between ${ {\mathcal F} }_{\varpi }({t}_{i})$ and ${ {\mathcal F} }_{\varpi }({t}_{j})$ rapidly accumulate, meaning that the pool of maximal simplexes shared by ${ {\mathcal F} }_{\varpi }({t}_{i})$ and ${ {\mathcal F} }_{\varpi }({t}_{j})$ rapidly thins out. After about 2 minutes (over 50 window shifts, |*i* − *j*| > 50) the difference is about 95% (Fig. 4B).

Since the coactivity complexes are induced from the pairwise coactivity graph *G* as clique complexes, we also studied the differences between the coactivity graphs at different moments of time by computing the normalized distance between the coactivity matrices (see Methods). The results demonstrate that the differences in *G*, i.e., between *G*(*t*_*j*_) and *G*(*t*_*i*_), accumulate more slowly with temporal separation than in ${ {\mathcal F} }_{\varpi }$: after about two minutes the connectivity matrices differ by about 10–15% (Fig. 4C).

Figure 4D shows the asymmetric distance between two consecutive coactivity complexes ${ {\mathcal F} }_{\varpi }({t}_{i})$ and ${ {\mathcal F} }_{\varpi }({t}_{i+1})$, and the asymmetric distance between the starting and a later point ${ {\mathcal F} }_{\varpi }({t}_{1})$ and ${ {\mathcal F} }_{\varpi }({t}_{i})$, normalized by the size of ${ {\mathcal F} }_{\varpi }({t}_{1})$ as a function of time. The results suggest that, although the sizes of the coactivity complexes at consecutive timesteps do not change significantly, the pool of the maximal simplexes in ${ {\mathcal F} }_{\varpi }$ is nearly fully renewed after about two minutes (see Suppl. Movie 1). In other words, although the coactivity complex changes its shape slowly, the integrated changes across long periods are significant (compare Fig. 4E with Fig. 2B). Biologically, this implies that the simulated cell assembly network, as described by the model, completely rewires in a matter of minutes (see Suppl. Movies 2 and 3).

**Figure 5:**
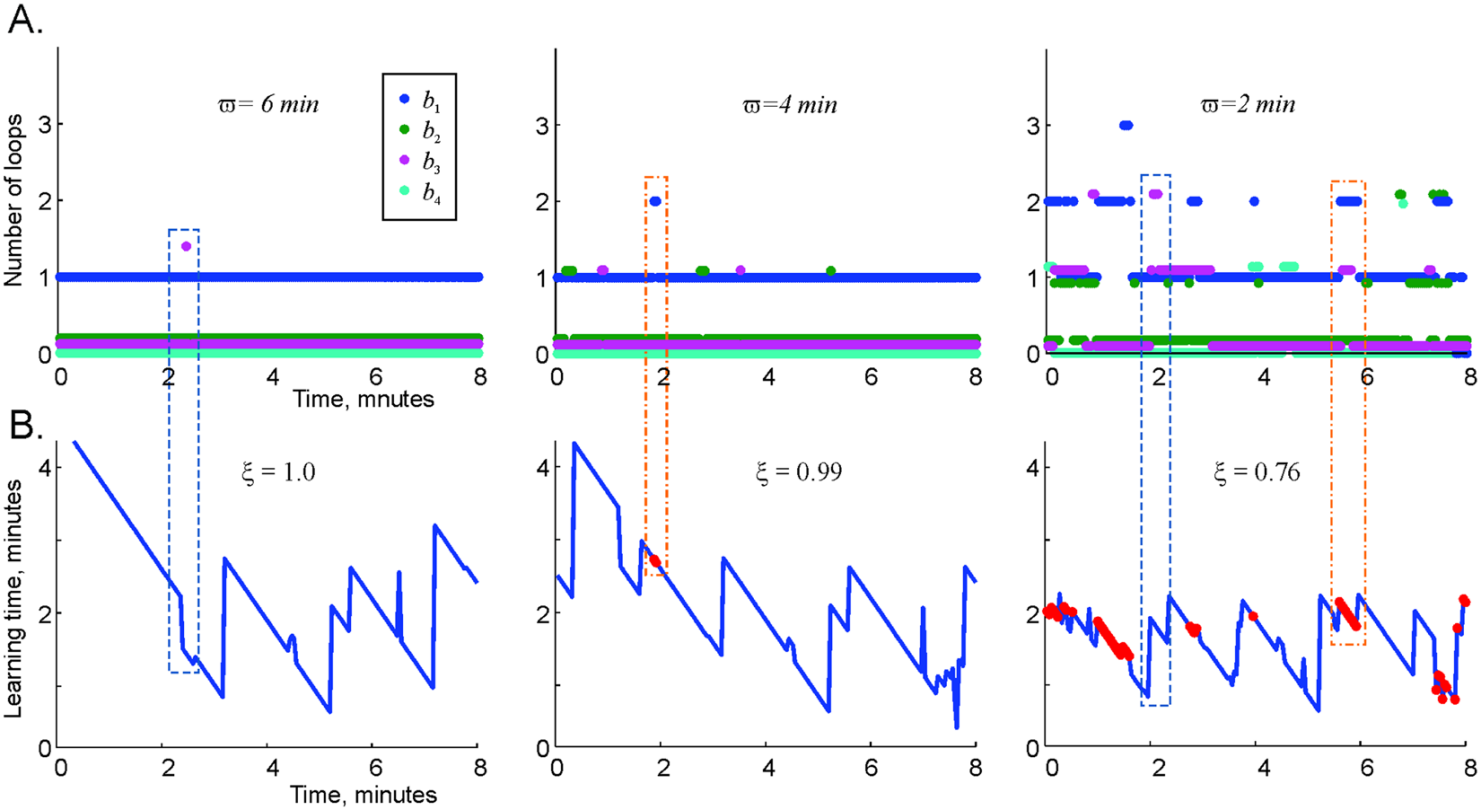
Stability of large-scale topological information. (**A**) The low-dimensional Betti numbers *b*_1_, *b*_2_, *b*_3_ as a function of discrete time, computed for three coactivity integration windows, *ϖ* = 6 minutes, *ϖ* = 4 minutes and *ϖ* = 2 minutes. The 0th Betti number, *b*_0_ = 1, remains stable at all times and is therefore not shown. At sufficiently large coactivity windows, $\varpi \sim 4-6$ minutes, the topological fluctuations become suppressed and the large-scale topological information remains stable, even though the characteristic lifetime of a maximal simplex in the coactivity complex ${ {\mathcal F} }_{\varpi }$ is about 10 secs (Fig. 4C). As the integration window narrows, the topological fluctuations intensify (Suppl. Fig. 2). (**B**) The variation in the time required to extract the topological information increases as the coactivity integration window shrinks. At *ϖ* = 6 min, there are no failing points. Notice that the starting learning time corresponds to the *T*_*min*_ value computed for the “perennial” case *ϖ* = ∞, illustrated on (Fig. 1B). As the memory window shrinks to *ϖ* = 4 min, the topological mapping fails in only 1% of the cases—just one point over 8 minute interval, when a spurious 1*D* loop appears. At *ϖ* = 2 min, the complex ${ {\mathcal F} }_{\varpi }$ fails to produce the correct topological information in 24% of the cases (convergence score *ξ* = 0.76). The failing moments are marked by red dots. Here we compute the most conservative estimate for the learning time *T*_*min*_ to be the time required to establish the correct topology only in the dimensions that may contain physical obstructions, 0*D* and 1*D*. Therefore, the points where *T*_*min*_ diverges are marked by appearances of spurious 1*D* loops (encapsulated into red dashed boxes across panels). The points where the learning time rapidly changes are often accompanied by the appearance or disappearance of higher dimensional topological loops (blue dashed boxes).

**Topological analysis of the flickering coactivity complex** exhibits a host of different behaviors. First, we start by noticing that the 0th and the higher-order Betti numbers always assume their physical values *b*_0_ = 1, *b*_*n*>4_ = 0, whereas the intermediate Betti numbers *b*_1_, *b*_2_, *b*_3_ and (for small *ϖ*s) *b*_4_ can fluctuate (Fig. 5A and Suppl. Fig. 3). In other words, despite the fluctuations of its simplexes, the flickering complex ${ {\mathcal F} }_{\varpi }$ does not disintegrate into pieces (i.e., *b*_0_ > 1 is never observed) and and produces no noncontractible topological loops in dimensions *D* > 4 (i.e., *b*_*n*>4_ = 0). Biologically, this implies that the topological fluctuations in the simulated hippocampal map are limited to 1*D* loops, 2*D* surfaces and 3*D* bubbles. For example, an occurrence of *b*_1_ = 2 value indicates the appearance of an extra (non-physical) 1*D* loop that surrounds a spurious gap in the cognitive map (Fig. 1A). On the other hand, at the moments when *b*_1_ = 0, all 1*D* loops in ${ {\mathcal F} }_{\varpi }$ are contractible, i.e., the central hole is not represented in the simulated hippocampal map^52^. The moments when *b*_*n*>2_ > 0 indicate times when the flickering complex ${ {\mathcal F} }_{\varpi }$ contains non-physical, non-contractible multidimensional topological surfaces. One can speculate about the biological implications of these fluctuations, as illustrated in Suppl. Fig. 5.

As the coactivity window increases, the fluctuating topological loops become suppressed and vice versa, as the integration window shrinks, the fluctuations of the topological loops intensify (Fig. 5). This tendency could be expected, since the cell assembly lifetimes reduce as the integration window shrinks and increase as the coactivity integration window grows (Fig. 3F). However, a nontrivial result suggested by Fig. 5 is that the topological parameters of the flickering complex can stabilize completely, even though its maximal simplexes keep appearing and disappearing, or “flickering.” At *ϖ* ≈ 6 minutes, the Betti numbers of ${ {\mathcal F} }_{\varpi }$ remain unchanged (Fig. 5A), whereas the lifetime of its typical simplex is about 10 seconds (Fig. 3F). Biologically, this implies that a stable hippocampal map can be encoded by a network of transient cell assemblies, i.e., that the ongoing synaptic plasticity in the hippocampal network does not necessarily compromise the integrity of the large-scale representation of the environment.

### Local learning times

If information about the detected place cell coactivities is retained indefinitely, the time required to produce the correct topological barcode of the environment *T*_*min*_ may be computed only once, starting from the onset of the navigation, and used as the low-bound estimate for the learning time^40, 41, 44^. In the case of a rewiring (transient) cell assembly network, the pool of encoded spatial connectivity relationships is constantly renewed. As a result, the time required to extract the large-scale topological signatures of the environment from place cell coactivity becomes time-dependent and its physiological interpretation also changes. *T*_*min*_(*t*_*k*_) now defines the period over which the topological information emerges from the ongoing spiking activity at every stage of the navigation, i.e., defines a local span of the learning period. Thus, the process of extracting the large-scale topology of the environment should be quantified in terms of the mean learning time *T*_*min*_ = 〈*T*_*min*_(*t*_*k*_)〉_*k*_ and its variance Δ*T*_*min*_/*T*_*min*_, which does not exceed 40% (typically Δ*T*_*min*_/*T*_*min*_ ≈ 20%). This suggests that *T*_*min*_ provides a statistically sound characteristic of the information flow across the simulated cell assembly network.

As shown in Fig. 5B, the proportion *ξ* of “successful” coactivity integration windows (those windows in which *T*_*min*_ assumes a finite value) depends on their width *ϖ*. For small *ϖ*, the coactivity complex frequently fails to reproduce the topology of the environment (Fig. 5A). As *ϖ* grows, the number of failing points, i.e., those for which *T*_*min*_(*t*_*k*_) > *ϖ* (red asterisks on Fig. 5A), reduces due to the suppression of topological fluctuations. Moreover, the domains previously populated by the divergent points are substituted with the domains of relatively high but still finite *T*_*min*_(*t*_*k*_). For sufficiently large coactivity windows (*ϖ* > 6 minutes), such divergent points become exceptional or disappear entirely: the correct topological information is recaptured within all memory windows.

Of note, the time dependence of *T*_*min*_(*t*_*k*_) exhibits abrupt increases and decreases, with characteristic 45° slants in-between. The rapid rises of *T*_*min*_(*t*_*k*_) correspond to appearances of obstructions in the coactivity complex ${ {\mathcal F} }_{\varpi }$ (and possibly higher-dimensional surfaces) that temporarily prevent certain spurious loops from contracting. As more connectivity information is supplied by the ongoing spiking activity, the coactivity complex ${ {\mathcal F} }_{\varpi }$ may acquire a combination of simplexes that eliminates these obstructions, allowing the unwanted loops to contract and yielding the correct topological barcode. Thus, Fig. 5B suggests that the dynamics of the coactivity complex is controlled by a sequence of coactivity events that produce or eliminate topological loops in ${ {\mathcal F} }_{\varpi }$, while the 45° slants in *T*_*min*_(*t*_*k*_) represent “waiting periods” between these events (since with each window shift over Δ*t*, the local learning time decreases by exactly the same amount).

**Figure 6:**
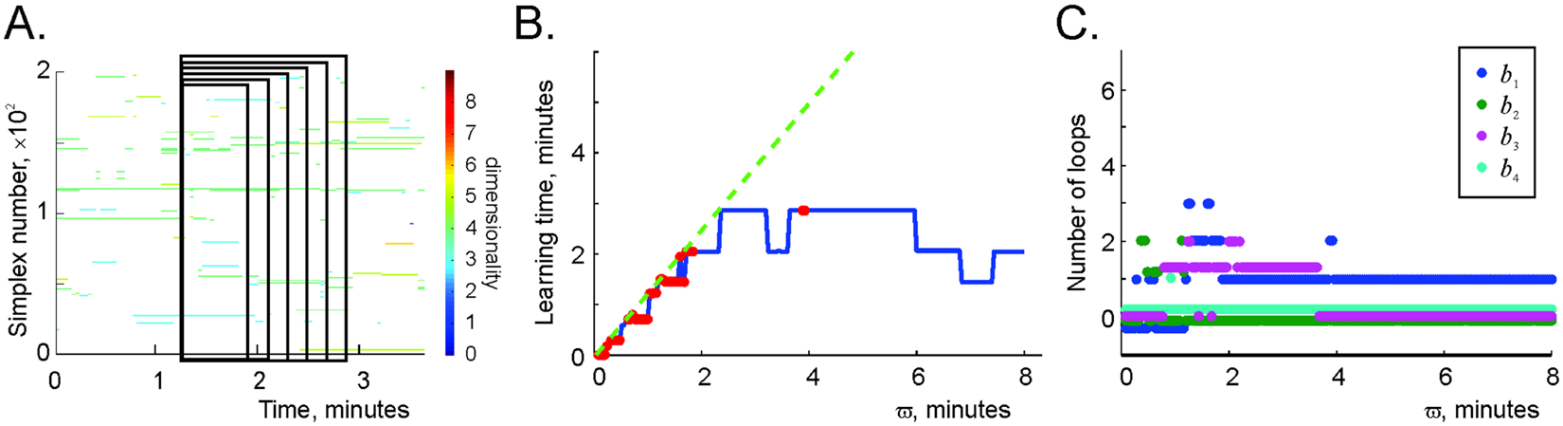
Stability the large-scale topological information. (**A**) A schematic illustration of the growing coactivity window *ϖ*, superimposed over a fragment of the maximal simplex’ timeline diagram on Fig. 4A. (**B**) The learning times *T*_*min*_(*t*_*k*_) computed within the growing coactivity window are shown by the blue line. For narrow coactivity windows, the learning times either diverge (*T*_*min*_(*t*_*k*_) > *ϖ*) or converge barely (*T*_*min*_(*t*_*k*_) ≈ *ϖ*). As *ϖ* exceeds a certain critical value *ϖ*_*c*_ (for the simulated place cell ensemble, *ϖ*_*c*_ ≈ 4–6 minutes), the learning time *T*_*min*_(*t*_*k*_) stops increasing and begins to fluctuate around a certain mean value *T*_*min*_ = 〈*T*_*min*_(*t*_*k*_)〉_*k*_. This value is independent of the coactivity window width and hence represents a parameter-free characterization of the mean time required to extract topological information from place cell coactivity. (**C**) The low-dimensional Betti numbers *b*_1_, *b*_2_, *b*_3_ and *b*_4_ as a function of the coactivity integration window width *ϖ*. As *ϖ* exceeds a critical value *ϖ*_*c*_, the Betti numbers *b*_*n*_ stabilize, indicating suppression of the topological fluctuations in ${ {\mathcal F} }_{\varpi }$.

To better understand how the learning time depends on the coactivity integration window width, we tested the dependence of *T*_*min*_ on *ϖ* by fixing the position of several coactivity integration windows *ϖ*_*k*_ and expanding their right side, ${\varpi }_{k}^{(1)} > {\varpi }_{k}^{(2)} > \ldots  > {\varpi }_{k}^{(q)}$ (Fig. 6 and Suppl. Fig. 4). As one would expect, small values of *ϖ* generated many failing points, whereas the learning times *T*_*min*_(*t*_*k*_) computed for the successful trials remained nearly equal to *ϖ*, i.e., the width of the narrow integration windows was barely sufficient for producing the correct barcode ${\mathfrak{b}}( {\mathcal E} )$. However, as *ϖ* grows further, *T*_*min*_ stops increasing and, as *ϖ* exceeds a certain critical value *ϖ*_*c*_ (typically about five or six minutes), the learning time begins to fluctuate around a mean value *T*_*min*_ = 〈*T*_*min*_(*t*_*k*_)〉 of about two minutes. In other words, for sufficiently large coactivity windows *ϖ* > *ϖ*_*c*_, the learning times become *independent* of the model parameter *ϖ*, and therefore the model provides a parameter-free characterization of the time required by a network of place cell assemblies to represent the topology of the environment, whereas *ϖ*_*c*_ defines the time necessary to collect the required spiking information (Suppl. Fig. 5).

## Discussion

Fundamentally, the mechanism of producing the hippocampal map depends on two key constituents: on the temporal relationships among the action potentials produced by the place cells and by the way in which spiking information is processed by the downstream networks. A key determinant for the latter is the synaptic architecture of the cell assembly network, which changes constantly due to various forms of synaptic and structural plasticity: place cell assemblies may emerge in cell groups that exhibit frequent coactivity or disband due to lack thereof. The latter phenomenon is particularly significant: since the hippocampal network is believed to be one of the principal memory substrates, frequent recycling of synaptic connections could compromise the integrity of its net function. For example, the existence of many-to-one projections from the CA3 to the CA1 region of the hippocampus suggests that the CA1 cells may serve as readout neurons for the assemblies formed by the CA3 place cells^8, 53^. Electrophysiological studies suggest that the recurrent connections within CA3 and the CA3–CA1 connections rapidly renew during the learning process and subsequent navigation^54, 55^. On the other hand, it is also well known that lesioning these connections disrupts the animal’s performance in spatial^56–58^ and nonspatial^59, 60^ learning tasks, which suggests that an exceedingly rapid recycling of functional cell groups impairs the formation of the hippocampal map^61–64^.

The proposed model allows us to investigate whether a dynamically rewiring network of place cell assemblies can sustain a stable topological representation of the environment. The results suggest that if the intervals between consecutive appearance and disappearance of the cell assemblies are short (or, in an alternative interpretation, if the readout neurons have short memory retention span), the hippocampal map exhibits strong topological fluctuations. However, if the cell assemblies rewire sufficiently slowly, the information encoded in the hippocampal map remains stable despite the transience of connections in its neuronal substrate. Thus, the plasticity of neuronal connections, which is ultimately responsible for the network’s ability to incorporate new information^65–68^, does not necessarily degrade the information that is already stored in the network. Moreover, Fig. 5 suggests that the network’s failure to produce a topological barcode at a particular moment (within a particular integration window *ϖ*_*k*_) is typically followed by a period of successful learning. This implies that the forgetting mechanism incorporated into the model, whereby the removal of older connectivity relationships from ${ {\mathcal F} }_{\varpi }$ as newer relationships are acquired, allows correction of some of the accidental connections that may have been responsible for producing persistent spurious loops at previous steps. In other words, a network capable of not only accumulating, but also forgetting information, exhibits better learning results. These results present a principal development of the model outlined in refs 40–42, 44 from both a computational and a biological perspective.

### Physiological vs. schematic learnings

The schematic approach proposed in ref. 23 allows us to describe the process of spatial learning from two perspectives: as training of the synaptic connections within the cell assembly network—referred to as physiological learning in ref. 23—or as the process of establishing large-scale topological characteristics of the environment, referred to as cognitive learning. The difference between these two concepts is particularly apparent in the case of the rewiring cell assembly network, in which the synaptic configurations may remain unsettled due to the rapid transience of the connections. On the other hand, cognitive learning is perfectly well defined since the large-scale topological characteristics of the environment can be achieved reliably.

In fact, the model outlines three spatial information processing dynamics at the short-term, intermediate-term, and long-term memory timescales^69^. First, local spatial connectivity is represented in transient cell assemblies within several seconds. This timescale corresponds to the scope of memory processes that involve temporary maintenance of information produced by the ongoing neural spiking activity, commonly associated with short-term memory^69, 70^. The short-term memory capacity is around seven (7 ± 2) items^71^, corresponding in the model to the order of the simulated cell assemblies (Fig. 3B). Information about the large-scale connectivity of the environment is acquired and updated at the timescale of the mean learning time *T*_*min*_ (Figs 5 and 6), at the order of minutes, corresponding to intermediate-term memory timescale^72, 73^. Persistent topological information, represented by the stable Betti numbers, may represent long-term memory about the connectivity of the environment as a whole.

## Methods

**The rat**’**s movements** were modeled in a small planar environment, similar to the arenas used in electrophysiological experiments (bottom of Fig. 1A). The trajectory simulates non-preferential exploratory behavior, without favoring of one segment of the environment over another. In particular, this allows us to avoid inducing artificial topological loops in the coactivity complexes.

**Place cell spiking activity** is modeled as a stationary temporal Poisson process with a spatially localized Gaussian rate characterized by the peak firing amplitude *f*_*c*_ and place field size *s*_*c*_^74^. The results are based on a simulated ensemble of *N*_*c*_ = 300 place cells, with log-normally distributed peak firing amplitudes (mode *f* = 14 Hz) and place field sizes (mode *s* = 17 cm). The place cell spiking probability is modulated by the *θ*-component of the extracellular field oscillations (mean frequency of ~8 Hz^75^) recorded in wild-type Long Evans rats (see Methods in ref. 17). These values, selected based on our previous studies of topological maps encoded by place cell ensembles, guarantee the existence of a correct topological map in a population of “perennial” cell assemblies (*ϖ* = ∞). For more computational details and a discussion of the range of behavioral and physiological parameters see refs 40, 41, 44.

**The activity vector** of a place cell *c* is constructed by binning its spike trains into an array of consecutive coactivity detection periods *w*. If the time interval *T* splits into *N*_*w*_ such periods, then the activity vector of a cell *c* over this period is *m*_*c*_(*T*) = [*m*_*c*;1_, …, *m*_*c*;*Nw*_], where *m*_*c*;*k*_ specifies how many spikes were fired by *c* into the *k*-th time bin^42^. The activity vectors of *N*_*c*_ cells, combined as rows of a *N*_*c*_ × *N*_*w*_ matrix, form the *activity raster R*. A *binary raster B* is obtained from the activity raster *R* by replacing the nonzero elements of *R* with 1.

**Place cell spiking coactivity** is defined as firing that occurs over two consecutive *θ*-cycles, which is an optimal coactivity detection period *w* both from the computational^41^ and from the physiological^48^ perspective. Coactivity *ρ* of a pair of cells *c*_1_ and *c*_2_ can be computed as the formal dot product of their respective activity vectors *ρ*_*c*1*c*2_ = *m*_*c*1_(*T*)*m*_*c*2_(*T*).

### Shifting coactivity window

The spiking activity confined within the *k*-th coactivity integration window of size *ϖ* produces a local binary raster *B*_*k*_ of size *N*_*c*_ × *N*_*ϖ*/*w*_, where ${N}_{\varpi /w}=\lfloor \varpi /w\rfloor$. The coactivity integration window was shifted by the discrete timesteps Δ*t* = 10*w* ≈ 2.5 s. Thus, in *n*_*s*_ = *ϖ*/Δ*t* steps, the local rasters *B*_*k*_ and *B*_*k* + *ns*_ cease to overlap. During the four-minute-long coactivity integration window *n*_*s*_ = 96.

Within each coactivity integration window *ϖ*_*k*_, the most frequently activated connections give rise to a local set of cell assemblies, which may replace some of the previously existing assemblies. The mean recycling rate of the cell assemblies is characterized by the decay constant *τ*_*ϖ*_.

### Coactivity distances

For each window *ϖ*_*n*_, we compute the coactivities of every pair of cells

1 $${\rho }_{ij}^{n}=\sum _{k}\,{B}_{{i}_{k}}^{n}{B}_{{j}_{k}}^{n},$$

where ${B}_{{i}_{k}}^{n}$ is the “local” binary raster of coactivities produced within that window. To compare different local rasters, we compute the similarity coefficients between them

2 $${r}_{mn}=\sum _{i\mathrm{.}j}\,|{\rho }_{ij}^{n}-{\rho }_{ij}^{m}|/\sum _{i\mathrm{.}j}\,|{\rho }_{ij}^{n}|,$$

where indexes *i*,*j* run over all the cells in the ensemble, illustrated in Fig. 3C.

**Topological analyses** were implemented using the JPlex package^76^.

## Electronic supplementary material

Supplementary Materials

Supplementary Movie 1.

Supplementary Movie 2.

Supplementary Movie 3.
