# Supplementary material for: Transient cell assembly networks encode stable spatial memories
Source: Sci Rep. 2017 Jun 21;7:3959. doi: 10.1038/s41598-017-03423-3 (PMC5479874; doi:10.1038/s41598-017-03423-3)
Supplement: Supplementary file 1 — Supplementary Materials [file 41598_2017_3423_MOESM1_ESM.pdf]

Transient cell assembly networks encode stable spatial memories

Andrey Babichev and Yuri Dabaghian

SUPPLEMENTARY FIGURES

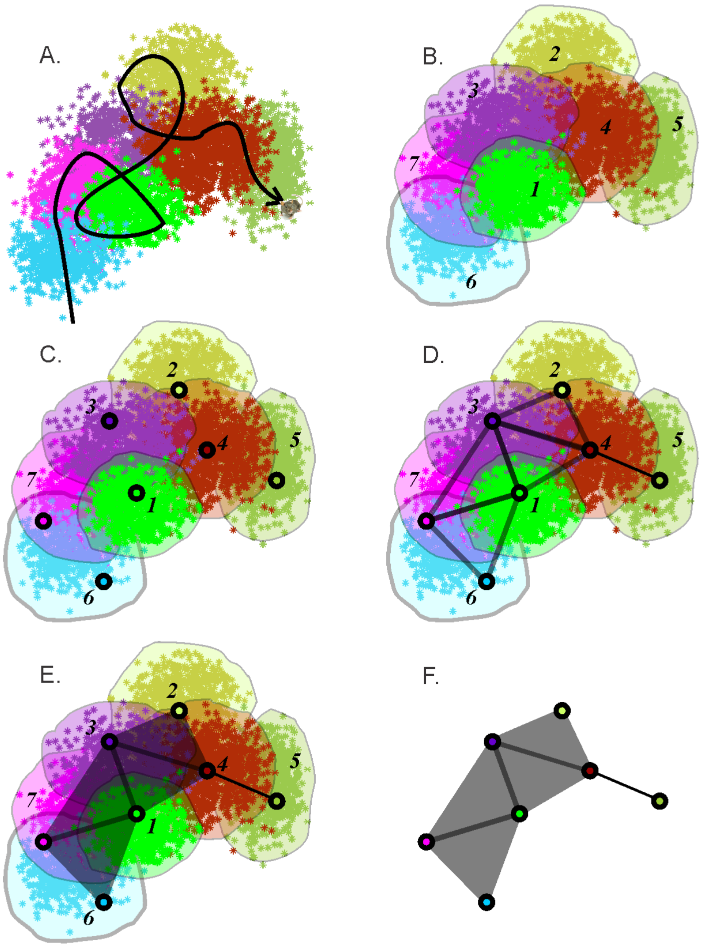

Supplementary Figure 1. An illustration of the Alexandrov-Čech theorem. (**A**) A spatial domain traversed by a short fragment of the simulated trajectory (black line). The rat's locations at the moments where seven simulated place cells produced their spikes are marked by asterisks of seven different colors. (**B**) The place fields (regions marked by ovals) form a cover of the environment. The construction of the nerve complex $\mathcal{N}$ is illustrated on the following panels. (**C**) Each element of the cover corresponds to a vertex of the nerve complex: vertexes are shown by small colored discs. (**D**) Every overlapping pair of place fields contributes a one-dimensional ($1D$) link to the nerve complex. The result is the $1D$ skeleton of $\mathcal{N}$. (**E**) Every triple of place fields that has common intersection contributes a two-dimensional ($2D$) facet (triangle), which together form the $2D$ skeleton of the nerve complex. (**F**) According the Alexandrov-Čech theorem, the $2D$ skeleton represents the topology of the cover shown on panel A.

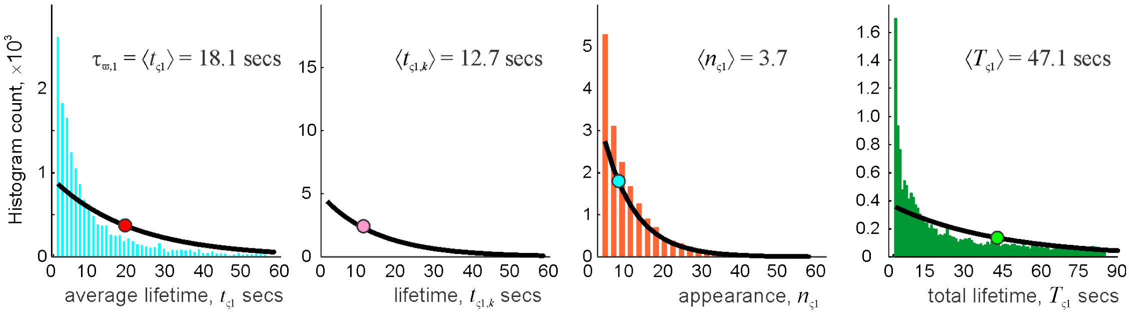

Supplementary Figure 2. The statistics for the flickering connections in the cells assemblies. The statistics of the mean lifetimes t_{ς_1}, individual lifetimes $t_{\varsigma_1,k}$, number of appearances n_{ς_1} and the total existence periods T_{ς_1} of the $1D$ subsimplexes of the coactivity complex $\mathcal{F}_{\varpi}$, which is the links of the coactivity graphs $G(\varpi_n)$, representing pairwise connections in the simulated cell assemblies. In all cases, the mean net existence period equals approximately to the product of the mean lifetime by the mean number of appearances $\langle T_{\varsigma_1}\rangle \approx \langle n_{\varsigma_1}\rangle\langle t_{\varsigma_1,k}\rangle$.

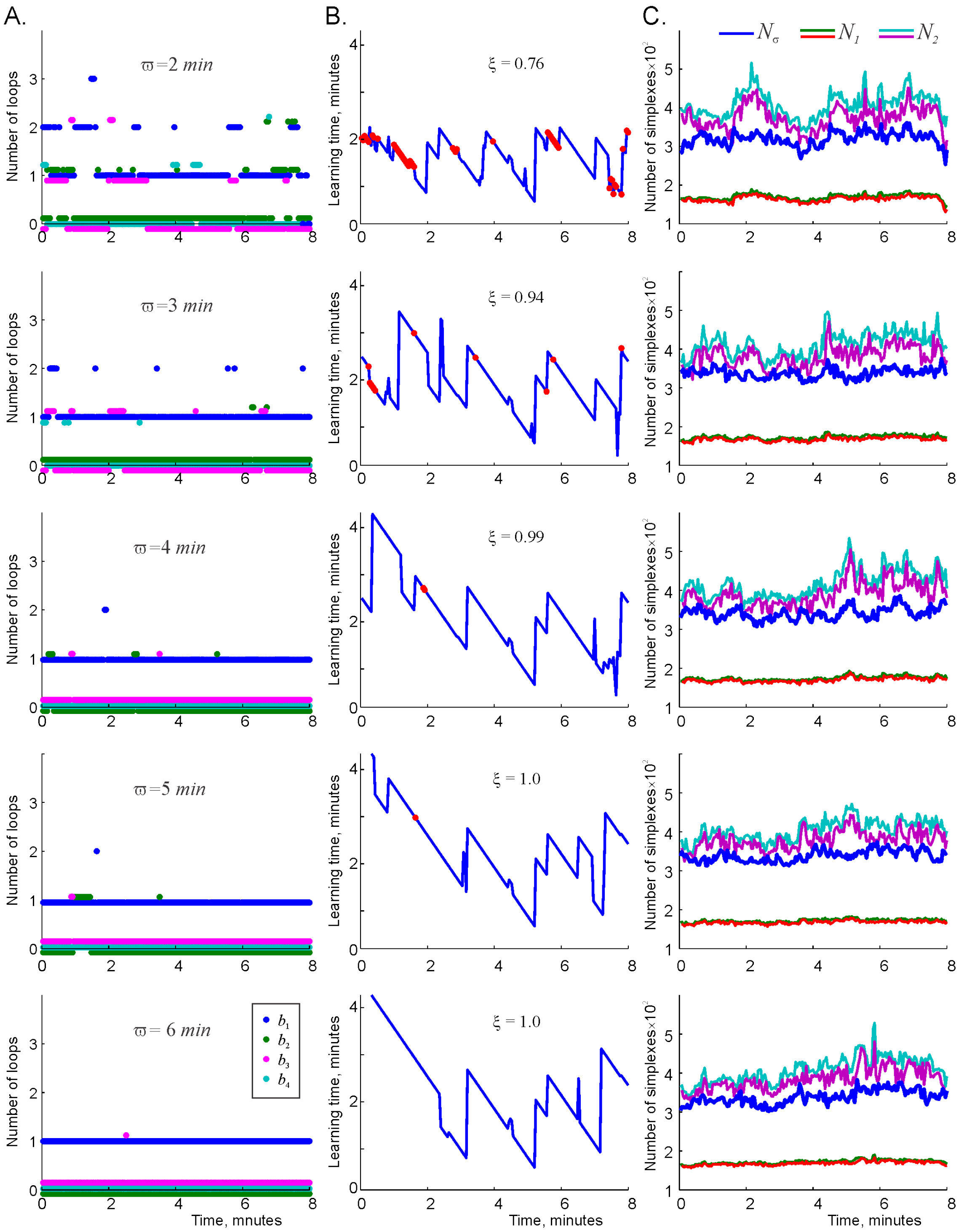

Supplementary Figure 3. Flickering coactivity complex as a function of time. (**A**) As the coactivity integration window ϖ increases, the topological fluctuations in the coactivity complex $\mathcal{F}_\varpi$ are suppressed. (**B**) The corresponding learning times T_{min}. Red dots mark the moments when the map acquires a non-physical topological barcode. As the coactivity window ϖ grows, the topological fluctuations are suppressed and the number of failures decreases. Notice that the learning time remains high immediately after the areas as the failures are suppressed. At $\varpi \approx 5$ min, when the mean half-life of a simulated cell assembly is about $\tau_\varpi \approx 10$ secs (Fig. 3D), the map retains a topologically correct shape at all times. (**C**) Variations in the size of the coactivity complex $\mathcal{F}_\varpi$ reduce with increasing ϖ.

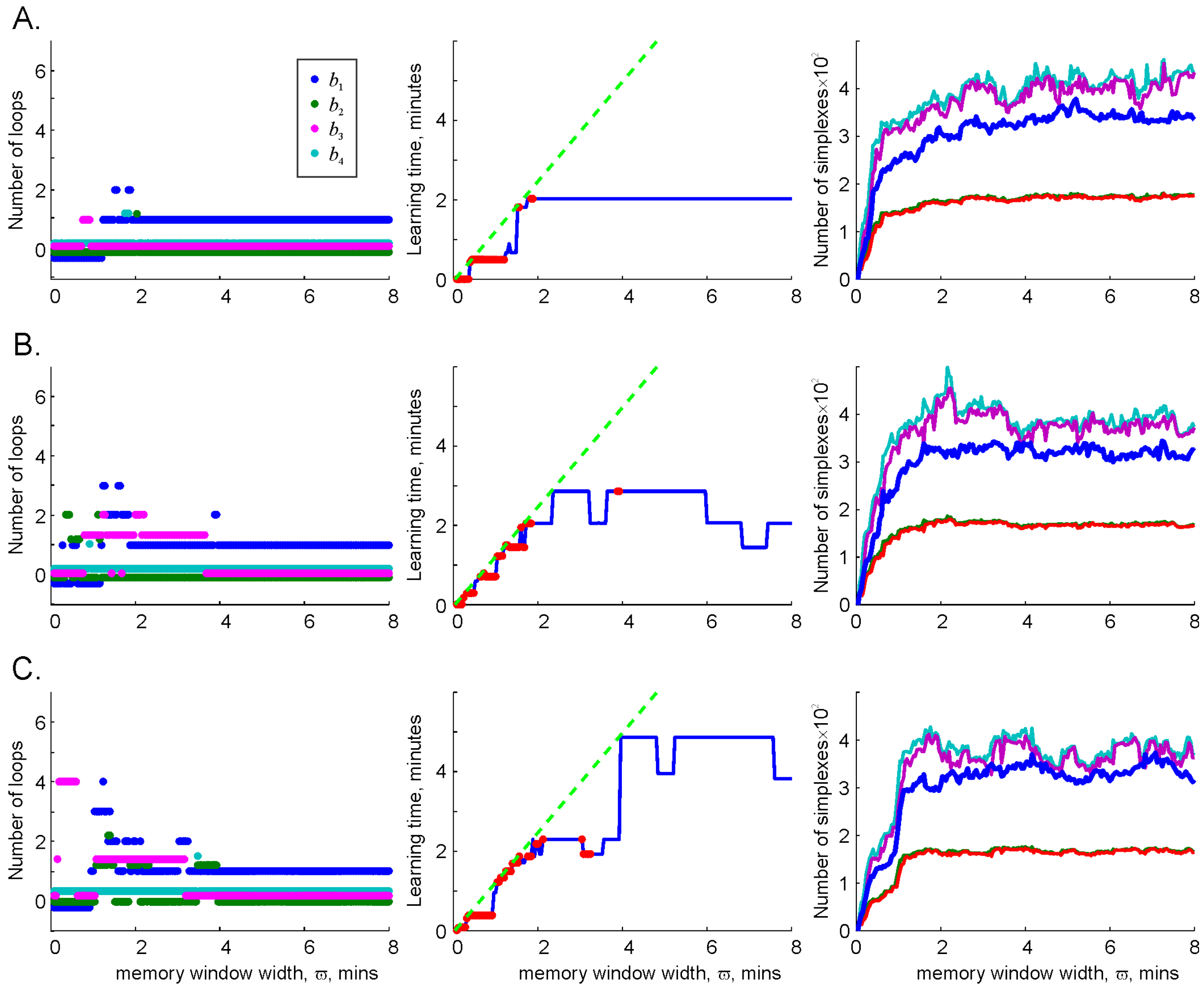

Supplementary Figure 4. Growing memory window. (**A**) If the coactivity window is placed at a time-point with few topological fluctuations, the Betti numbers b_1, b_2, b_3 and b_4, and the learning time quickly stabilize. The last panel indicates that size of the coactivity complex $\mathcal{F}_{\varpi}$ grows as a function of ϖ and then acquires a stable size. (**B**) At a typical temporal domain, the behavior of the "asymptotic" coactivity complex $\mathcal{F}_{\varpi}$ exhibits stronger topological fluctuations and the learning time fluctuates as a function of increasing ϖ. (**C**) In the locations in which the topological fluctuations are strong, the Betti numbers of the flickering coactivity complex $\mathcal{F}_{\varpi}$ take longer to stabilize and the learning time may retain high values for longer periods, before returning to the typical regime shown on panel B.

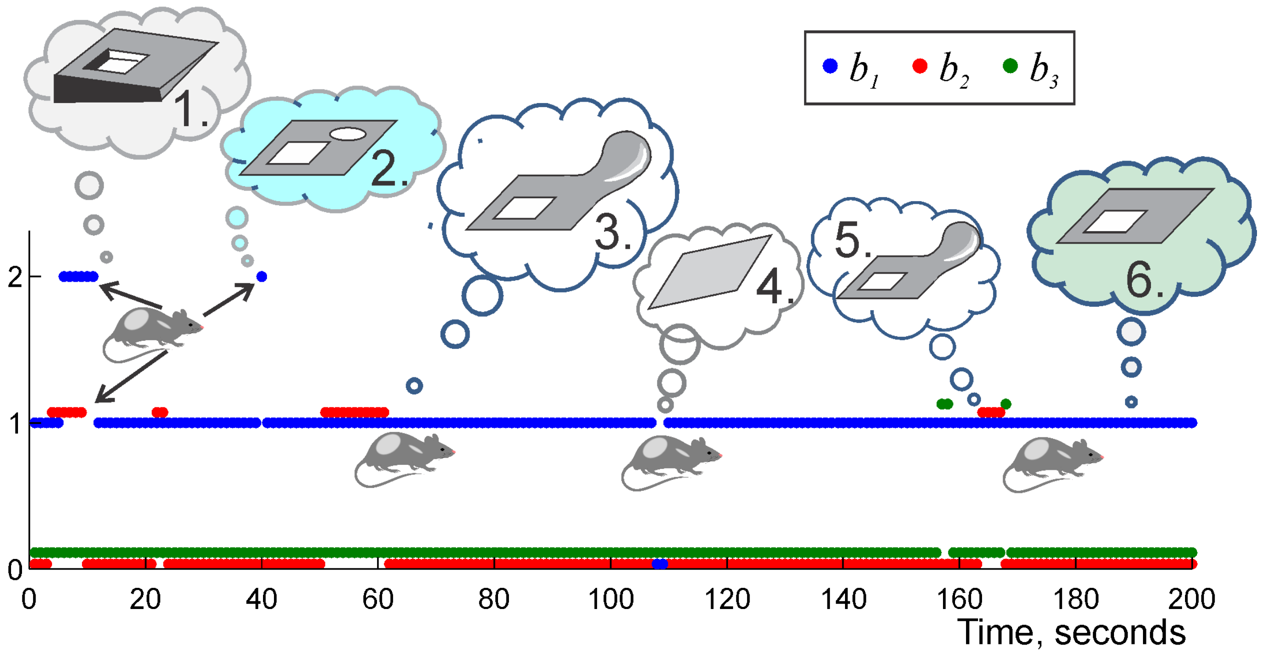

Supplementary Figure 5. Topological fluctuations of the hippocampal map. As in the case illustrated in Fig. 5, we assume that the map forms one single piece at all times, hence its 0-th Betti number $b_0 = 1$ is not shown. At the moments when the Betti numbers assume values $b_1 = 2$, $b_2 = 1$ and $b_{n>2} = 0$, the hippocampal map is topologically equivalent to a torus, similar to the one shown on Fig. 1C. Alternatively, $\mathcal{F}_\varpi$ can be pictured as the surface of the "thickened" environment $\mathcal{E}$. At the moments when $b_1 = 2$ and $b_{n>1} = 0$, the map thins back into a $2D$ sheet and acquires an extra $1D$ loop indicating an extra (spurious) gap in $\mathcal{F}_\varpi$. At the times when $b_1 = b_2 = 1$ and $b_{n>2} = 0$, the map contains a non-contractible $2D$ loop and a non-contractible cycle; one can picture a hollow bulge appearing on the side of the environment $\mathcal{E}$. If $b_{n>0} = 0$, the map degenerates into a contractible $2D$ sheet with no holes, i.e., at these moments the information about the central hole in $\mathcal{E}$ is lost. However, for most times the topological barcode of $\mathcal{F}_\varpi$ coincides with the topological barcode of the simulated environment, $b_1(\mathcal{F}_\varpi) = b_1(\mathcal{E}) = 1$ and $b_{n>1}(\mathcal{F}_\varpi) = b_{n>1}(\mathcal{E}) = 0$ (see Fig. 1C).

SUPPLEMENTARY MOVIE CAPTIONS

Supplementary Movie 1. Coactivity integration window sliding over the spike raster. The dark ticks in the background represent spikes arranged along the time axis. Each row of spikes corresponds to a particular place cell. On the right, the green dots mark the locations where the cell assemblies, formed during the first coactivity integration window ϖ_1, have ignited. The blue dots represent the cell assembly fields at the subsequent timesteps. As the coactivity window shifts, green dots disappear, indicating that the original population of the cell assemblies is replaced by newly forming ones.

Supplementary Movie 2. A network of transient cell assemblies. The place cells (dark dots) produce synaptic connections (straight lines) to the downstream readout neurons (small circles). Changes of the coactivity patterns of the simulated place cell groups result in formation of new cell assemblies and disbanding of some old ones (Fig.2A). On average, the simulated cell assembly network contains about $N_{\varsigma} = 320$ cell assemblies. The color of the connections represents the order, $|\varsigma|$, of the cell assemblies.

Supplementary Movie 3. Illustrative examples of the rewiring cell assemblies. The connections in about 100 of randomly selected, transient cell assemblies are shown in horizontal projection into the navigated environment.
